# Genetic Diversity of Endospore-forming Nitrogen-fixing Bacteria and Their Future Application as Biofertilizers in the Central Dry Zone of Myanmar

**DOI:** 10.1264/jsme2.ME25033

**Published:** 2025-12-13

**Authors:** Ya Maon Phoo, Koki Toyota, Yu Yu Min

**Affiliations:** 1 Graduate School of Bio-Applications and Systems Engineering, Tokyo University of Agriculture and Technology, 2–24–16, Naka-cho, Koganei, Tokyo 184–8588, Japan; 2 Department of Agricultural Microbiology, Yezin Agricultural University, Nay Pyi Taw, 15013, Myanmar

**Keywords:** *Paenibacillus*, *Priestia*, *Bacillus*, acetylene reduction assay, agricultural waste

## Abstract

In Myanmar, the application of both nitrogen-based chemical fertilizers and biofertilizers is limited and this low input has caused poor agricultural yields. The present study aimed to isolate indigenous endospore-forming nitrogen-fixing bacteria (EFNFB) and examine their potential for co-inoculation with agricultural waste. A total of 387 isolates were obtained from 42 different soil samples in the central dry zone of Myanmar using nitrogen-free Rennie medium. Nitrogen-fixing activity (NFA) assessed with the acetylene reduction assay was positive in 102 isolates. A phylogenetic ana­lysis based on 16S rRNA sequences identified 25 different species, including the genera *Paenibacillus*, *Priestia*, *Bacillus*, *Brevibacillus*, *Sporolactobacillus*, *Niallia*, and *Neobacillus*. Among these genera, *Paenibacillus* spp. was the predominant genus, comprising 51 isolates (64%) across 16 different species (64%) that were prevalent in soils rotated with rice and pulses. *Paenibacillus* spp. showed different NFA levels in Rennie medium. Eleven species belonging to different genera had not been previously documented as nitrogen-fixing bacteria. NFA levels were evaluated in soil inoculated with EFNFB and rice straw or mung bean residue. The results obtained demonstrated that NFA levels were dependent on isolates and the type of agricultural waste. NFA in soil was significantly increased by inoculations with some isolates, suggesting their potential as biofertilizers. The inoculation of *Priestia aryabhattai* S10 with rice straw or mung bean resulted in significantly higher NFA levels in soil. These results indicate the potential of EFNFB as biofertilizer inoculants in Myanmar.

Nitrogen (N) is a major essential nutrient in crop production and is important for maintaining and improving crop growth and yield (Orchardson, 2020). Although the application of chemical N fertilizers enhances crop growth and yield, high rates may diminish the quality of agricultural products (Liu *et al.*, 2019). In addition, the long-term excessive use of chemical fertilizers has resulted in serious negative impacts, such as soil hardening, acidification, and environmental pollution (Jiao *et al.*, 2018). Therefore, a decrease in reliance on chemical fertilizers is expected for sustainable management and a reduction in their environmental impact (Alves *et al.*, 2004). Biological nitrogen fixation (BNF) is attracting increasing attention as such an approach (Choudhury and Kennedy, 2004) and the use of efficient inoculants is regarded as an important strategy.

In Myanmar, legumes are important cash crops and approximately 70% of all legume crops are grown on 4.2–4.3 million ha during the winter season (October to January), with yields ranging between 1.0–1.3 t ha^–1^ (GAIN, 2020), which is markedly lower than those in Vietnam (14.3‍ ‍t‍ ‍ha^–1^) and Thailand (12.2 t ha^–1^). Lower yields in Myanmar are attributed to inappropriate crop management practices and insufficient technology (Aye *et al.*, 2013). In‍ ‍Myanmar, most farmers do not use chemical fertilizers due to their high price. According to the FAO (2021), Myanmar’s fertilizer consumption was only 38.7‍ ‍kg ha^–1^, compared to 139‍ ‍kg ha^–1^ in Thailand and 193‍ ‍kg ha^–1^ in India. The average N fertilizer application rate in Myanmar for pulses is estimated to be only 10‍ ‍kg of N ha^–1^, which is markedly lower than 90–170‍ ‍kg ha^–1^ for Thailand and Vietnam (MAPSA, 2023). Therefore, N input other than chemical fertilizers may be needed to increase crop yields in Myanmar.

Crop residue return may increase yields and quality by improving soil organic matter, soil physical properties, water use efficiency, and soil structural stability and reducing soil bulk density (Soon and Lupwayi, 2012; Li *et al.*, 2019). However, previous studies reported a negative impact on both the environment and crop yields (Lu, 2020). The decomposition of crop residues causes N starvation, which is not conducive to crop growth and yields (Wang *et al.*, 2018). We interviewed farmers in Myanmar and found that many returned crop residues, such as rice straw and pulse straw, directly or after burning. Burning is more practical due to its low cost and feasibility, and enables fields to be cleared quickly for the next planting and also avoids N starvation. Some farmers consider burning to control pests and enhance soil fertility; however these benefits are limited and short-lived. While a small number of farmers are aware of the advantages of crop residue return, this practice alone is not sufficient to obtain better crop yields. Therefore, a technology that stimulates the soil N status is expected, and biofertilizers are attracting increasing attention (Zhao *et al.*, 2024).

One type of biofertilizer is a substance containing living microorganisms, such as *Rhizobium* and *Trichoderma*, which are applied to seeds, plant surfaces, or soil (Vessey, 2003). The use of biofertilizers has been shown to improve soil fertility by fixing atmospheric N, solubilizing insoluble phosphates, and/or producing plant growth-promoting substances in the soil (Mazid and Khan, 2015). In Myanmar, the use of biofertilizers is not widely practiced. Although some local products are available, *e.g.*, *Trichoderma* and *Rhizobium*, the amount produced does not cover the whole agricultural area (Herridge *et al.*, 2008). Although some commercial products, including *Bacillus* sp., are imported from Thailand and China, indicating prospects for yield increases by inoculation technology, most farmers cannot afford them due to their high costs. To address this issue, it is necessary to isolate indigenous microorganisms as an alternative biofertilizer for Myanmar’s agriculture.

Endospore-forming bacteria, such as *Bacillus* spp., *Paenibacillus* spp., and *Brevibacillus* spp., are major soil bacteria (Alexander, 1977). Several strains of *Bacillus* spp. exhibit antibacterial and antifungal activities against phytopathogens and, thus, may function as biocontrol agents (Fira *et al.*, 2018). In addition, some endospore-forming bacteria have the ability to fix atmospheric N. For example, more than 30 members of *Paenibacillus* spp. have been isolated as N fixers from soils and rhizospheres and include *Paenibacillus polymyxa*, *Paenibacillus macerans*, *Paenibacillus peoriae*, *Paenibacillus azotofixans*, and *Paenibacillus stellifer* (Wang *et al.*, 2013; Li *et al.*, 2022). Among *Bacillus* spp., approximately 10 have the ability to fix atmospheric N, and *Bacillus cereus*, *Bacillus pumilus*, *Bacillus circulans*, *Bacillus licheniformis*, and *Bacillus subtilis* have been isolated from soils and rhizospheres (Ding *et al.*, 2005). The genus *Priestia* used to belong to the genus *Bacillus* and some species fix N, such as *Priestia endophytica*,* Priestia megaterium*, and *Priestia aryabhattai* (Sharma *et al.*, 2022; Liu *et al.*, 2023; Pal *et al.*, 2025). In the genus* Niallia*, *Niallia circulans* fixes N (Hashem *et al.*, 2019). In the genus *Brevibacillus*, *Brevibacillus agri* fixes N (Suriani *et al.*, 2022) and further studies are needed to assess this capability in the other species of this genus.

Bacteria survive for a long time after they form an endosphere due to resistance to extreme environmental conditions, drying, pH, and salinity in soil (Logan and De Vos, 2009). They then actively colonize the rhizosphere where they establish beneficial interactions with plants, indicating their potential contribution to BNF (Xie *et al.*, 2016). Therefore, the present study focused on N-fixing bacteria that form endospores because they may enhance the soil N status and be readily applied to fields as an inoculum for biofertilizers.

In Myanmar, the screening of indigenous endospore-forming N-fixing bacteria (EFNFB) and their application as biofertilizers have not yet been exami­ned. The study of effective indigenous EFNFB and feasible application methods with waste products represents a sound approach for future sustainable agriculture. Therefore, the aims of the present study were to screen indigenous EFNFB in Myanmar soil, examine the effects of bacterial inoculations on N-fixing ability in the soil, and propose how waste products may be managed by inoculations with effective indigenous EFNFB as biofertilizers.

## Materials and Methods

### Isolation of bacteria from Myanmar soils

**Fig. 1.**
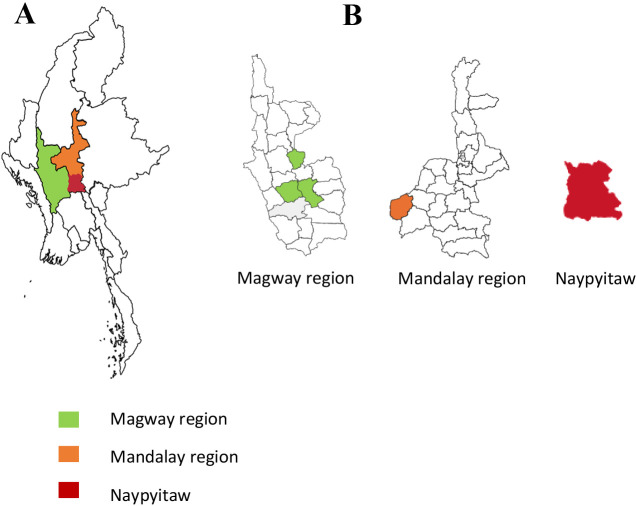
The central dry zone (Magway, Mandalay, and Naypyitaw) of Myanmar (A) and soil collection sites in each region (B)

Soil samples were collected from different cultivated fields (sesame, groundnut, green gram, cowpea, pigeon pea, chickpea, black gram, cranberry bean, cluster bean, rice bean, lima bean, hyacinth bean, winged bean, and yard long bean) in different regions (Magway, Mandalay, and Nay Pyi Taw), Myanmar (Fig. 1). Soil was sampled from five different locations in each field and a composite soil sample was made. Soil samples were sieved through a 5-mm mesh sieve and mixed well. Composite soil samples (100 g) were air dried and kept at room temperature (27°C).

Ten grams of each soil sample was added to a 200-mL Erlenmeyer flask containing 90‍ ‍mL of sterile tap water to make a 10^–1^ dilution. It was then heated in a water bath for 10‍ ‍min at 80°C to obtain endospore-forming bacteria (Bendt, 1985). The flask was immediately transferred into a water bath. One-hundred microliters each of serially diluted suspensions (10^–1^, 10^–2^, 10^–3^, and 10^–4^) was inoculated into a vial containing semi-solid (0.3% agar) N-free Rennie medium (Elbeltagy *et al.*, 2001).

A turbid medium a few days to one week after the inoculation indicated the presence of N-fixing bacteria. The turbid part was streaked onto several plates including N-free Rennie medium with 1.5% agar. Bacterial colonies of different shapes and colors on each plate were considered to be different strains and were separately purified on fresh solid N-free Rennie medium.

16S rRNA genes were amplified using extracted DNA as a template with the universal primers 27f (5′-AGAGTTTGATCMTGGCTCA-3′) (Frank *et al.*, 2008) and 1378r (5′-CGGTGTGTACAAGGCCCGGGACG-3′) (Heuer *et al.*, 1999). DNA extraction was conducted using the method reported by Miyashita (1992) and PCR amplification was performed using an Ex premier DNA polymerase (Takara Bio) with the conventional method (Jamily *et al.*, 2018). PCR products were purified with a FavorPrepTM GEL/PCR Purification Mini Kit (Chiyoda Science) and were sent for sequencing at FASMAC. Eighty isolates were sequenced for *ca.* 800 bp using the 1378r primer. Regarding 8 isolates (F1, F2, F4, F12, F14, S3, S10, and S12) showing low similarity values to the closest species, an additional *ca.* 800 bp were sequenced using the primer 27f and the resulting sequences were aligned and combined using ClustalW v2.1. The resulting 16S rRNA sequences were analyzed using BLAST against the DDBJ/ENA/GenBank database (https://blast.ncbi.nlm.nih.gov/Blast.cgi), applying a 98.7% sequence similarity threshold as the species-level cut-off (Kim *et al.*, 2014). Sequences were deposited in the DDBJ/ENA/GenBank database under accession numbers LC872246 to LC872325. A phylogenetic tree was constructed in MEGA v12 (Kumar *et al.*, 2024) using the maximum likelihood (ML) method based on *ca.* 800 bp of 16S rRNA gene sequences aligned with ClustalW, applying the general time reversible (GTR) nucleotide substitution model and 1,000 bootstrap replicates.

### Confirmation of nitrogen-fixing activity (NFA) with the acetylene reduction assay (ARA)

To confirm the NFA of bacterial isolates, ARA was conducted (Knowles and Barraquio, 1994). Three milliliters of semi-solid N-free Rennie medium was added to a 13-mL vial. One loopful of a bacterial isolate suspended in sterilized saline water (0.85% NaCl) was inoculated into the vial. The vial was closed with a butyl rubber stopper and incubated at 30°C for 24 h. Before the incubation, 1‍ ‍mL of the headspace air was removed and replaced with an equal volume of pure acetylene gas to create a 10% acetylene atmosphere. The ethylene concentration was measured after 1 and 7 days with a Shimadzu GC-14B gas chromatograph (Shimadzu) equipped with a hydrogen flame ionization detector (FID). The amount of ethylene in the headspace was quantified and activity was expressed as 3 different NFA levels: low (0–0.99‍ ‍nmol C_2_H_4_ culture^–1^ h^–1^), middle (1–9.99‍ ‍nmol C_2_H_4_ culture^–1^ h^–1^), and high (>10‍ ‍nmol C_2_H_4_ culture^–1^ h^–1^).

### Evaluation of NFA in soil

Mung bean residue and rice straw, which are typical rotation crops in the central dry zone in Myanmar, were used to examine the effects of the crop residue on N-fixing ability. Triplicate 30-mL vials were prepared, each containing 5‍ ‍g of air-dried soil (1.4‍ ‍g‍ ‍N‍ ‍kg^–1^, C/N ratio 9.5). Then, 15‍ ‍mg of each crop residue powder (rice straw: 7.7‍ ‍g N kg^–1^, 44.2 C/N ratio; mung bean: 12‍ ‍g‍ ‍N‍ ‍kg^–1^, 34.7 C/N ratio) was placed on the soil surface. Tap water and the bacterial suspension were then added to the soil surface in order to adjust its moisture content to 60% of the maximum water holding capacity (MWHC) and its bacterial density to 1×10^7^‍ ‍CFU (g soil)^–1^. Bacterial suspensions were prepared by an overnight culture in 10^–1^ strength of Difco^TM^ nutrient broth. Vials were covered with aluminum foil and incubated at 30°C. After 6 days, vials were sealed with a rubber septum and 10% of the headspace was then replaced with pure acetylene gas. The vials were incubated again at 30°C for 24‍ ‍h and ethylene was measured as described above. After the ethylene measurement, the rubber septum was replaced with aluminum foil and the incubation was continued at 30°C. Measurements were conducted periodically at 7-day intervals four times and the moisture content was adjusted to 60% MWHC every week. Rice straw was used in Exp. 1 and 2 and mung bean residue in Exp. 3 to examine the effects of different crop residues on the efficiency of EFNFB in the soil. Although different isolates were used in each experiment at different times, the inoculation method, incubation conditions, and ARA measurement procedures were consistent across all experiments. Results were expressed as the cumulative NFA for ethylene in 1 month.

### Statistical ana­lysis

The N-fixing ability of EFNFB in the soil with crop residue was compared to the control (-), and the significance of differences was analyzed using the Student’s *t*-test (IBM SPSS Statistics 20) and Microsoft Excel (ver. 2111).

## Results

### Isolation of endospore-forming nitrogen-fixing bacteria and their NFA

**Fig. 2.**
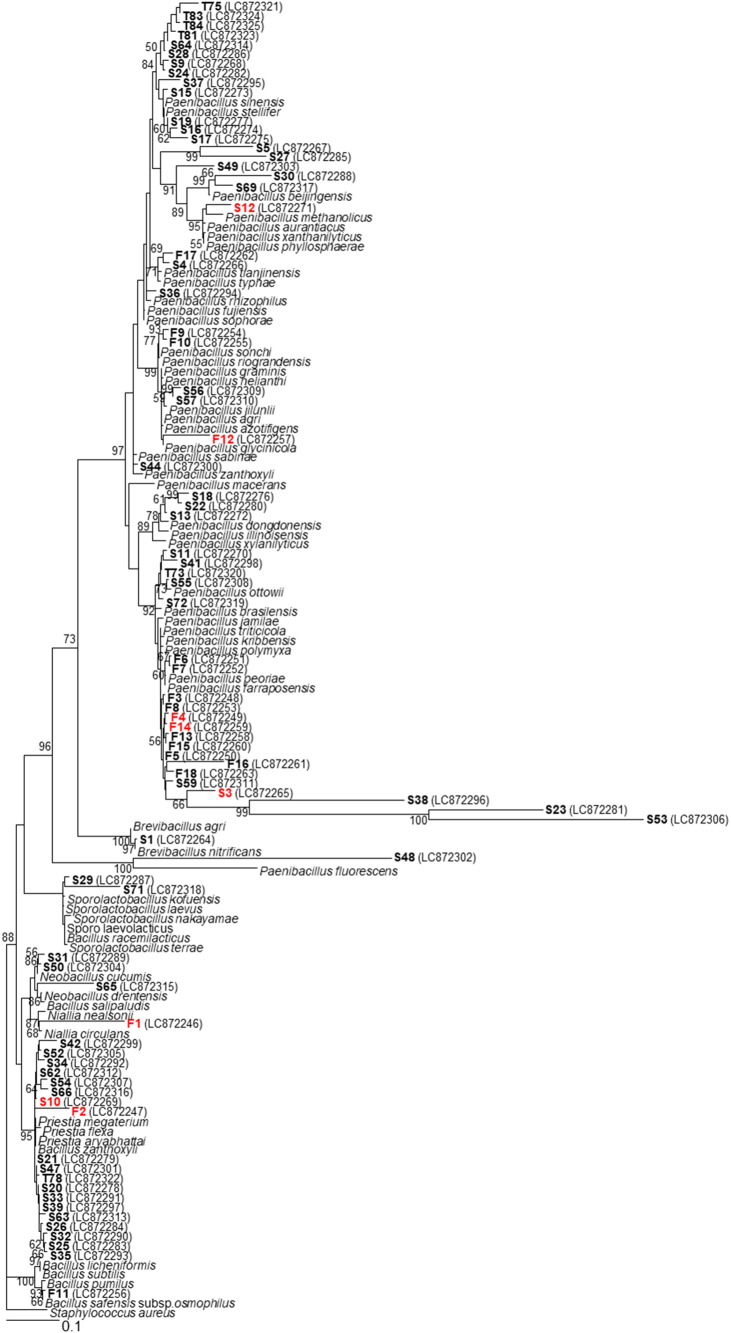
Phylogenetic tree based on 16S rRNA gene sequences, drawn using 800 bp from the reverse primer. Stains shown in red were sequenced for 1,400 bp. Reanalyzed results with BLAST are shown in Table 1. The scale bar indicates 10% sequence divergence. Reference strains were obtained from the DDBJ database. *Staphylococcus aureus* was used as the outgroup. Bootstrap value ≥50%.

A total of 387 isolates were isolated from 42 soil samples cultivated with different crops in different regions. Of these, 102 isolates exhibited NFA in ARA. Among the 102 NFA-positive isolates, 90 were identified at the genus level. These included 10 non-spore-forming bacteria, such as *Azospirillum* spp., *Pandoraea* spp., *Burkholderia* spp., and *Microbacterium* sp. The remaining 80 isolates were endospore-forming bacteria, *i.e.*, the genera *Paenibacillus* (64%), *Priestia* (26%), *Bacillus* (3%), *Sporolactobacillus* (3%), *Neobacillus* (3%), *Brevibacillus* (1%), and *Niallia* (1%) (Table 1 and Fig. 2).

### Diversity of endospore-forming nitrogen-fixing bacteria

The genus *Paenibacillus* was prevalent across different NFA levels (Table 1). Specifically, the strain with the highest NFA level belonged to the genus *Paenibacillus*. In the middle range of NFA isolates, *Paenibacillus* accounted for 68%, followed by *Priestia* (32%). Among the low NFA strains, *Paenibacillus* was present at 49%, followed by other genera, such as *Priestia* (29%), *Bacillus* (6%), *Sporolactobacillus* (6%), *Neobacillus* (6%), *Niallia* (3%), and *Brevibacillus* (3%).

Fifty-one isolates were the closest to the genus *Paenibacillus* and were distributed across 16 distinct species. *Pa. stellifer* (9 isolates), *Pa. polymyxa* (8 isolates), and *Pa. peoriae* (4 isolates) were frequently found in soil samples and were prominent among high NFA isolates. The remaining 13 *Paenibacillus* species were less frequent across different NFA levels. Among them, *Paenibacillus kribbensis* exhibited the highest NFA level, followed by *Pa. polymyxa*. Only two isolates (3%) were the closest to *Bacillus* (*Bacillus safensis* and *Bacillus salipaludis*), both of which showed low NFA levels. *Priestia* was also frequently detected, with 21 isolates (26%) across *Pr. megaterium* (11 isolates) and *Pr. aryabhattai* (6 isolates), both of which exhibited middle and low NFA levels.

A single species was detected in the genera *Brevibacillus* (*Brevibacillus nitrificans*) and *Niallia* sp. Additionally, two isolates were the closest to the genera *Neobacillus* and *Sporolactobacillus*. These 4 genera showed low NFA levels.

The majority of isolates belonged to the genus *Paenibacillus* spp., and *Pa. stellifer* (9 strains), the dominant species in this genus, had a similarity range of 98.8 to 99.9% (Table 1). Isolates closest to *Priestia* spp. were related to *Pr. aryabhattai* (99.5–100%) and *Pr. megaterium* (99.1–100%). *Bacillus* spp. isolates showed the highest similarity to *Ba. safensis* (99.8%) and *Bacillus salipaludis* (99.9%). *Neobacillus* spp. isolates were very similar to *Neobacillus drentensis* (99.9%) and *Neobacillus cucumis* (99.5%). The *Sporolactobacillus* spp. isolate was the closest to *Sporolactobacillus laevolacticus* (>98.8%) and that of *Brevibacillus* sp. was the closest to *Br. nitrificans* (99.8%).

### Effects of cropping patterns on the diversity of endospore-forming nitrogen-fixing bacteria

Among the 80 isolates, 36 were found in soils under rice-pulses rotations and 18 were predominantly detected in a pulses-sesame rotations (Table 2). Additionally, 11 isolates were identified in pulses-chili rotations, 9 in pulses-onion rotations, 5 in rice-sesame rotations, and 1 in pulses-only soil. Within the rice-pulses rotation, *Paenibacillus* (53%) and *Priestia* (39%) were the most frequently isolated genera, followed by *Neobacillus* (6%) and *Sporolactobacillus* (3%). In the pulses-sesame crop rotation, *Paenibacillus* was‍ ‍the dominant genus (83%), while *Bacillus*, *Priestia*, and‍ ‍*Niallia* were each detected at 6%. Additionally, *Paenibacillus* spp. occupied 89% of isolates from pulses-onion rotations, 64% from pulses-chili rotations, and 40% from rice-sesame rotations.

Furthermore, most isolates from groundnut and black gram cultivated soils were *Paenibacillus* spp. *Priestia* spp. were mainly isolated from groundnut fields and less frequently from sesame, black gram, pea, and cluster bean soils. *Ba. safensis* and *Ba. salipaludis* were isolated from sesame and pea cultivated fields, respectively. *Neobacillus* spp. were isolated from groundnut soils under the rice-pulses rotation in the Naypyitaw region. Two *Sporolactobacillus laevolacticus* strains were isolated from groundnut and chickpea cultivated soils. *Niallia* sp. was isolated from sesame soil and *Br. nitrificans* from pigeon pea cultivated soil. N-fixing isolates from groundnut and sesame soils exhibited the highest NFA levels across various genera.

### Evaluation of nitrogen-fixing ability in the soil

N-fixing ability was higher in soil inoculated with any bacterial isolate than in the control without an inoculation (Table 3). Additionally, the rice straw amendment increased NFA in some isolates. In Exp. 1, *Pa. peoriae* F13 exhibited a significant (*P*<0.05) increase in NFA upon the addition of rice straw. Conversely, *Pa. polymyxa* F4 and *Paenibacillus* sp. F16 did not exhibit marked changes in NFA, regardless of the presence or absence of the residue. In Exp. 3, *Paenibacillus typhae* S4 and *Pr. aryabhattai* S10 showed a significant (*P*<0.05 and *P*<0.01) increase in NFA upon the addition of rice straw. In the absence of crop residues, *Pr. megaterium* F2, *Paenibacillus sonchi* F10, *Paenibacillus* sp. S27, and *Pa. typhae* S4 all exhibited significantly higher NFA levels (*P*<0.01 and *P*<0.05). When combined with mung bean residue, *Pa. polymyxa* F6 and *Pr. aryabhattai* S10 exhibited significant (*P*<0.05 and *P*<0.01) increases in NFA. *Pr. aryabhattai* S10 also showed a significantly (*P*<0.01) higher NFA level in the soil upon the addition of rice straw or mung bean residues than that with organic amendment. There was no correlation between the NFA of isolates in N-free media in vials (Table 1) and in soil (Table 3).

## Discussion

This is the first study to isolate EFNFB from soils cultivated with different crops in Myanmar and examine their application as biofertilizers. To isolate EFNFB, soil samples were heat-treated at 80°C for 10‍ ‍min (Bendt, 1985). Molecular identification showed that 89% of the isolates were endospore-forming bacteria (Fig. 2), while 11% did not form spores. These results suggest that the heat treatment method was not perfect, but effectively selected endospore-forming bacteria, similar to previous studies. *Paenibacillus* spp. were the predominant genus (Table 1), which is consistent with previous findings showing that *Paenibacillus* spp. are ubiquitous in nature and capable of forming resistant endospores (Bloemberg and Lugtenberg, 2001). Many studies have already reported that some endospore-forming bacteria, *e.g.*, *Bacillus* spp. and *Paenibacillus* spp., exhibit NFA (Ding *et al.*, 2005; Li *et al.*, 2022). The present results confirmed the role of endospore-forming bacteria in N fixation and their potential as biofertilizers.

The high abundance of EFNFB in the rice-pulses rotation and pulses-sesame rotation may be due to the favorable conditions created by legume cultivation. Legumes fix atmospheric N through symbiosis and enhance soil microbial diversity and activity and the establishment of free-living N-fixing bacteria (Qiao *et al.*, 2024). In addition, rice cultivation often involves temporary flooding, which creates an anaerobic environment in the soil (Wang *et al.*, 2021), whereas pulses restore aerobic conditions during their growth cycle. This rotation creates alternating soil redox conditions and may promote the survival of facultative anaerobes, such as *Paenibacillus* (Kiran *et al.*, 2017) and *Neobacillus* (Patel and Gupta, 2020; Schober *et al.*, 2025). In the present study, *Paenibacillus* was the predominant genus and was highly prevalent in soils associated with rice and pulses-based crop rotations (Table 2). In addition, two strains of *Neobacillus*, *N. cucumis* and *N. drentensis*, were isolated from soils under the rice-pulses crop rotation (Table 2). Strains belonging to these two facultative anaerobic genera may survive well under anaerobic conditions.

In the present study, most of the EFNFB identified have already been reported as N-fixing bacteria (Wang *et al.*, 2013; Li *et al.*, 2022; Liu *et al.*, 2023; Pal *et al.*, 2025). To the best of our knowledge, the NFA of 11 isolates, *i.e.*, *Pa.‍ ‍kribbensis*, *Pa. typhae*, *Paenibacillus triticicola*, *Paenibacillus xylanilyticus*, *Paenibacillus jamilae*, *Ba. salipaludis*, *Ba. safensis*, *Br. nitrificans*, *N. cucumis*, *N. drentensis*, and *S. laevolacticus*, have not been reported, although previous studies documented their beneficial activities for plants, such as enhancing nutrient uptake, providing biocontrol against pathogens, and promoting plant growth. Among them, *Pa. kribbensis*, with the highest NFA, has been associated with enhanced plant disease resistance against *Rhizoctonia solani* and tobacco mosaic virus (Canwei *et al.*, 2020) and phosphate-solubilizing ability (Marra *et al.*, 2012). *Pa. xylanilyticus* with a high NFA level was proposed as a chitin-degrading bacterium by Liao *et al.* (2019), which showed its potential in biotechnology, pharmaceuticals and phosphate solubilization (Pandya *et al.*, 2015). *Pa. jamilae* is known for its highly antagonistic activity against soil-borne pathogens and for its ability to increase beneficial bacteria in the wheat rhizosphere (Wang *et al.*, 2019). The results obtained herein newly demonstrated its high NFA. This study also identified a strain belonging to* Br. nitrificans*, which was previously proposed as a novel nitrifying bacterium species by Takebe *et al.* (2012), as a N fixer.

In the evaluation of NFA using soil, some strains exhibited increased NFA upon the addition of rice straw or mung bean (Table 3). Among them, *Pr. aryabhattai* S10 exhibited enhanced NFA following the addition of both rice straw and mung bean residue. This result indicates that *Pr. aryabhattai* S10 possessed the ability to effectively utilize different types of plant materials, which resulted in enhanced NFA. *Pr. aryabhattai* BPR-9 has been shown to produce extracellular enzymes, such as cellulases, amylases, pectinases, proteases, and lipases, facilitating the breakdown of complex polysaccharides in plant residues (Shahid *et al.*, 2022). Additionally, the whole-genome sequencing of *Pr. aryabhattai* strain S2 revealed the presence of genes involved in plant growth promotion, such as indole-3-acetic acid synthesis, and in salinity stress resistance, further supporting its adaptability in various environmental conditions (Sliti *et al.*, 2023). These findings indicate the potential of *Pr. aryabhattai* S10 as a biofertilizer for enhancing nitrogen input in rice-pulses crop rotation systems, particularly under residue management practices.

The addition of crop residues has been shown to enhance soil fertility and crop growth and change soil microbial diversity (He *et al.*, 2024). Therefore, inoculating straw with efficient microorganisms may enhance N fixation more effectively than an inoculation only (Roper and Ladha, 1995). The NFA of *Pr. aryabhattai* S10 and *Pa. peoriae* F13 increased following the addition of rice straw or mung bean residue. This result indicates that the isolates competed with indigenous microbes and utilized the crop residues, resulting in increased NFA. NFA increased over the incubation period, possibly because the residues gradually decomposed, providing more nutrients for bacteria. In contrast, the results of other isolates did not change. Some strains, such as *Pa. sonchi* F10 and *Paenibacillus* sp. S27, had higher NFA in the bacterial inoculation without the crop residue. In soil, inoculants must compete with the native population and the addition of a crop residue may increase competition. Isolates that showed lower NFA in the presence of the crop residue were considered to be less competitive with the crop residue. These results support previous findings showing that straw-associated N fixation was affected by many environmental and management factors (Roper and Ladha, 1995). It currently remains unclear whether the response of NFA to the addition of rice straw or mung bean differed depending on strains. Therefore, further research is needed to evaluate NFA by EFNFB in different soil types, various organic residues, and inoculation strategies in order to obtain a more detailed understanding of their effects on N fixation in the soil. We plan to use these EFNFB in rice-legume rotation fields to improve crop yields in Myanmar.

## Citation

Phoo, Y. M., Toyota, K., and Min, Y. Y. (2025) Genetic Diversity of Endospore-forming Nitrogen-fixing Bacteria and Their Future Application as Biofertilizers in the Central Dry Zone of Myanmar. *Microbes Environ ***40**: ME25033.

https://doi.org/10.1264/jsme2.ME25033

## Figures and Tables

**Table 1.** Nitrogen-fixing activities of 80 endospore-forming bacterial isolates from cultivated soils in Myanmar and their similarity percentages to closest species

NFA levels	Isolates	Closest species	Similarity %	C_2_H_4_ culture^–1^ h^–1^	NFA levels	Isolates	Closest species	Similarity %	C_2_H_4_ culture^–1^ h^–1^
**High**	S59	***Paenibacillus kribbensis***	99.8	42.5	**Middle**	F6	*Paenibacillus polymyxa*	99.8	1.1
	T73	*Paenibacillus polymyxa*	99.9	33.2		F17	***Paenibacillus typhae***	99.0	1.0
	S11	***Paenibacillus jamilae***	99.6	31.6		S25	*Priestia aryabhattai*	99.8	1.0
	F4	*Paenibacillus polymyxa*	99.8	28.4		S34	*Priestia megaterium*	99.6	1.0
	F3	*Paenibacillus peoriae*	99.8	19.1		F9	*Paenibacillus sonchi*	99.5	1.0
	S22	***Paenibacillus xylanilyticus***	99.9	19.0	**Low**	S4	***Paenibacillus typhae***	99.6	0.8
	S23	*Paenibacillus* sp.	81.2	17.6		F10	*Paenibacillus sonchi*	99.9	0.7
	F14	*Paenibacillus peoriae*	99.9	17.3		F11	***Bacillus safensis***	99.8	0.6
	S38	*Paenibacillus* sp.	85.9	17.1		S56	*Paenibacillus jilunlii*	99.0	0.6
	S17	*Paenibacillus* sp.	97.8	16.9		S27	*Paenibacillus *sp.	92.6	0.5
**Middle**	S15	*Paenibacillus sinensis*	99.3	8.5		T81	*Paenibacillus stellifer*	99.4	0.5
	F5	*Paenibacillus polymyxa*	99.8	6.2		S57	*Paenibacillus jilunlii*	99.3	0.5
	S66	*Priestia megaterium*	99.7	6.1		S12	*Paenibacillus* sp.	97.1	0.5
	F16	*Paenibacillus* sp.	94.3	5.2		S41	*Paenibacillus* sp.	98.6	0.4
	S9	*Paenibacillus stellifer*	99.6	5		F18	***Paenibacillus triticicola***	99.6	0.4
	S28	*Paenibacillus stellifer*	99.9	4.7		S55	*Paenibacillus polymyxa*	100.0	0.3
	T75	*Paenibacillus sinensis*	99.9	4.4		S39	*Priestia aryabhattai*	100.0	0.3
	S44	*Paenibacillus sabinae*	99.4	4.4		S33	*Priestia megaterium*	99.9	0.2
	F13	*Paenibacillus peoriae*	99.4	4.1		F1	*Niallia* sp.	96.4	0.2
	F12	*Paenibacillus* sp.	96.5	3.6		S72	*Paenibacillus polymyxa*	99.8	0.2
	S36	*Paenibacillus sophorae*	99.4	3.3		S42	*Priestia* sp.	96.9	0.1
	S24	*Paenibacillus stellifer*	99.9	3		S69	*Paenibacillus beijingensis*	99.4	0.1
	S10	*Priestia aryabhattai*	100	2.9		S1	***Brevibacillus nitrificans***	99.8	0.1
	T78	*Priestia aryabhattai*	99.9	2.9		S54	*Priestia megaterium*	99.1	0.1
	S21	*Priestia megaterium*	100	2.7		S65	***Neobacillus cucumis***	99.5	0.1
	S5	*Paenibacillus* sp.	93.1	2.6		S18	***Paenibacillus xylanilyticus***	99.3	0.1
	S20	*Priestia megaterium*	100	2.2		S63	*Priestia megaterium*	99.1	0.1
	S13	*Paenibacillus pabuli*	99.8	2.1		S30	*Paenibacillus* sp.	93.9	0.05
	T83	*Paenibacillus stellifer*	99	2.1		S62	*Priestia aryabhattai*	99.6	0.05
	S53	*Priestia* sp.	81.2	1.9		S29	***Sporolactobacillus laevolacticus***	99.6	0.05
	S19	*Paenibacillus stellifer*	99.8	1.8		S64	*Paenibacillus stellifer*	99.6	0.04
	F8	*Paenibacillus polymyxa*	99.5	1.7		S37	*Paenibacillus* sp.	97.9	0.04
	T84	*Paenibacillus stellifer*	99.5	1.5		S47	*Priestia megaterium*	100.0	0.03
	F15	*Paenibacillus peoriae*	99.9	1.4		S52	*Priestia megaterium*	99.9	0.02
	F7	*Paenibacillus polymyxa*	99.9	1.3		S71	***Sporolactobacillus laevolacticus***	98.8	0.02
	S35	*Priestia megaterium*	99.6	1.3		S48	*Priestia* sp.	82.0	0.02
	F2	*Priestia* sp.	98	1.3		S49	*Paenibacillus mucilaginosus*	99.5	0.01
	S3	*Paenibacillus* sp.	96.4	1.2		S32	*Priestia aryabhattai*	99.9	0.01
	S16	*Paenibacillus stellifer*	98.8	1.2		S31	***Neobacillus drentensis***	99.9	0.01
	S26	*Priestia megaterium*	99.5	1.2		S50	***Bacillus salipaludis***	99.9	0.01

Three different nitrogen-fixing activity levels: low (0–0.99‍ ‍nmol C_2_H_4_ culture^–1^ h^–1^), middle (1–9.99‍ ‍nmol C_2_H_4_ culture^–1^ h^–1^), and high (>10‍ ‍nmol C_2_H_4_ culture^–1^ h^–1^). Shaded letters indicate isolates belonging to *Paenibacillus* spp. Bold letters indicate isolates exhibiting nitrogen-fixing activity for the first time in this study.

**Table 2.** Sources of 80 bacterial isolates from cultivated soils under different crop rotations in this study

Closest species	No. of isolates	Rice-pulses	Rice-sesame	Pulses-sesame	Pulses-onion	Pulses-chili	Pulses
*Paenibacillus*							
*Pa. stellifer*	9	3	1		3	2	
*Pa. polymyxa*	8	2	1	5			
*Pa. peoriae*	4			4			
*Pa. sinensis*	2	2					
*Pa. xylanilyticus*	2	2					
*Pa. typhae*	2			1	1		
*Pa. sonchi*	2			2			
*Pa. sabinae*	1	1					
*Pa. jilunlii*	2					2	
*Pa. beijingensis*	1	1					
*Pa. triticicola*	1			1			
*Pa. sophorae*	1	1					
*Pa. pabuli*	1	1					
*Pa. mucilaginosus*	1					1	
*Pa. jamilae*	1				1		
*Pa. kribbensis*	1	1					
*Paenibacillus* sp.	15	5		2	3	2	
*Priestia*							
*Pr. megaterium*	11	9	1			1	
*Pr. aryabhattai*	6	4	1		1		
*Priestia* sp.	4	1	1	1		1	
*Bacillus*							
*Ba. safensis*	1			1			
*Ba. salipaludis*	1					1	
*Neobacillus*							
*N. cucumis*	1	1					
*N. drentensis*	1	1					
*Sporolactobacillus*							
*S. laevolacticus*	2	1				1	
*Brevibacillus*							
*Br. nitrificans*	1						1
*Niallia*							
*Niallia* sp.	1			1			
Total	80	36	5	18	9	11	1

**Table 3.** One-month cumulative nitrogen-fixing activities of endospore-forming bacteria inoculated to Tatkon soil in Myanmar with rice straw or mung bean crop residue (0.3%)

			C_2_H_4_ produced (n mol^–1^ day^–1^ [g soil]^–1^)		
	Isolates	NFA levels in N-free media	No Residue	+Rice straw	+Mung bean
Exp. 1	No inoculation		0	0.29±0.45	NT
	F4 (*Paenibacillus polymyxa*)	high	0.71±0.61	0.71±0.58	NT
	F13 (*Paenibacillus peoriae*)	middle	1.22±1.08	1.75±0.58*	NT
	F14 (*Paenibacillus peoriae*)	high	0.2±0.25	0.53±0.19	NT
	F16 (*Paenibacillus* sp.)	middle	0.58±0.41	0.53±0.38	NT
Exp. 2	No inoculation		0	0.38±0.66	NT
	S3 (*Paenibacillus* sp.)	middle	3.89±0.92	2.69±0.06	NT
	S9 (*Paenibacillus stellifer*)	middle	1.66±1.27	0.56±0.96	NT
	S11 (*Paenibacillus jamilae*)	high	2.44±1.08	0	NT
	S59 (*Paenibacillus kribbensis*)	high	1.50±1.41	2.92±3.49	NT
	S64 (*Paenibacillus stellifer*)	low	2.09±1.06	1.69±0.42	NT
	S66 (*Priestia megaterium*)	middle	0.29±0.51	1.22±1.59	NT
	S69 (*Paenibacillus beijingensis*)	low	1.13±1.30	1.84±1.39	NT
	S72 (*Paenibacillus polymyxa*)	low	0.92±1.60	0.46±0.80	NT
	T78 (*Priestia aryabhattai*)	middle	1.92±1.69	2.93±1.68	NT
Exp. 3	No inoculation		0	0.10±0.18	0.19±0.33
	F1 (*Niallia circulans*)	low	1.31±0.60	1.20±0.63	0.27±0.23
	F2 (*Priestia megaterium*)	middle	0.76±0.29*	1.24±0.86	0.41±0.36
	F6 (*Paenibacillus polymyxa*)	middle	0.43±0.75	0.20±0.35	0.55±0.21*
	F10 (*Paenibacillus sonchi*)	low	2.16±0.19**	1.38±1.28	0.44±0.39
	S4 (*Paenibacillus typhae*)	low	1.08±0.06**	1.53±0.80*	1.28±1.16
	S10 (*Priestia aryabhattai*)	middle	0.26±0.44	1.86±0.15**	1.44±0.23**
	S12 (*Paenibacillus* sp.)	low	1.19±1.11	0.26±0.44	0.29±0.26
	S27 (*Paenibacillus* sp.)	low	1.64±0.19**	0.62±1.07	0.23±0.21

This experiment was repeated three times using different isolates (Exp. 1, 2, and 3) and soil samples were incubated at 60% of the maximum water holding capacity. Nitrogen-fixing activity in soil represents mean values and the standard deviation of triplicate measurements. Significant differences between the inoculated treatments (bacteria, residues, and bacteria + residues) and control (no bacteria or residues) were exami­ned with a given comparison (independent samples *t*-test: * *P*<0.05, ** significant at *P*<0.01). NT: Not tested.
